# HIV-1 acquired drug resistance to integrase inhibitors in a cohort of antiretroviral therapy multi-experienced Mexican patients failing to raltegravir: a cross-sectional study

**DOI:** 10.1186/s12981-020-0262-y

**Published:** 2020-02-10

**Authors:** Aurelio Orta-Resendiz, Roberto A. Rodriguez-Diaz, Luis A. Angulo-Medina, Mario Hernandez-Flores, Luis E. Soto-Ramirez

**Affiliations:** Molecular Virology Unit, Infectious Diseases Department, Instituto Nacional de Ciencias Médicas y Nutrición Salvador Zubirán, Vasco de Quiroga 15, Belisario Dominguez, Tlalpan, P.O. Box 14080, Mexico City, Mexico

**Keywords:** Drug resistance, Raltegravir, Integrase inhibitors, Cross-resistance, Multi-experienced, Sequencing

## Abstract

**Background:**

In resource-limited settings, multi-experienced HIV infected patients are often prescribed raltegravir for salvage therapy. Patients failing raltegravir-containing regimens require other drugs including other integrase inhibitors. In this context, real-life data about the resistance and cross-resistance pathways between integrase inhibitors is limited. The aim of this study was to investigate integrase resistance pathways in a cohort of Mexican multi-experienced patients failing of a raltegravir-containing salvage regimen.

**Methods:**

Twenty-five plasma samples from subjects failing antiretroviral regimens which included raltegravir were obtained from various healthcare centres from 2009 to 2017 in Mexico. Antiretroviral history and demographics were collected. Samples were processed for integrase resistance genotyping testing by sequencing. The viral sequences were analysed with the Stanford HIV drug resistance database algorithm. Data was analysed with SPSS Statistics software.

**Results:**

We found a mean viral load of 4.17 log^10^ c/mL (SD 1.11) at the time of virologic failure. Forty-eight percent of the samples were raltegravir resistant. The Y143R/H/C substitutions were the most prevalent, followed by the N155H, and both Q148H/K and G140S/A in the same proportion. The Q148 + G140 combination was found in (12%) of the samples. Cross-resistance to elvitegravir was found in 83.3% and in 18.2% for both dolutegravir and bictegravir. Thirteen samples (52%) were susceptible to the four integrase strand-transfer inhibitors.

**Conclusions:**

Our findings suggest a high occurrence of resistance and cross-resistance to other integrase inhibitors among multi-experienced subjects failing raltegravir. We found a modestly lower proportion of cross-resistance to dolutegravir than data from clinical trials. Likely this drug could be used for salvage therapy. Explanations for the absence of mutations in half of the samples, other than reduced adherence, should be further investigated. Close surveillance is needed.

## Background

Antiretroviral therapy (ART) has increased the survival and reduced the morbidity of patients with HIV infection [1–4]. However, a major concern is the emergence of resistance-associated mutations (RAMs) that reduce the response to treatment [5–7]. Viral strains from patients that are not successfully treated frequently select RAMs. This makes the selection of a new ART regimen more difficult and increases the risk of virologic failure. Resistance genotyping studies are used to determine whether or not the viral strains are susceptible to a given drug [7], however, these tests are frequently not available in resource-limited settings.

The integrase strand-transfer inhibitors (INSTIs) are the most recent class of antiretroviral drugs approved for HIV infection [8]. These drugs work through the active inhibition of the integrase enzyme, which promotes the integration of the viral genome into the host DNA.

In the context of middle and low income-countries where integrase inhibitors are not widely available, these drugs were mostly used for salvage therapy in ART-experienced patients. In Mexico and most countries in Latin America, experience with integrase resistance and cross-resistance is limited.

Most patients treated with integrase inhibitors are using raltegravir (RAL), elvitegravir (EVG) or dolutegravir (DTG). Resistance and cross-resistance among these have been described in several studies (most of them in first-line therapy studies) that found RAMs related to the viral fitness and the potential decrease of response to salvage therapy [8–13]. This brings new challenges to the selection of second line and salvage therapies.

RAL has been the most commonly prescribed in our setting and has been used as salvage therapy for highly ART-experienced patients. RAL failing cases are limited and integrase resistance testing is not widely available [14]. Besides the data from clinical trials, there is limited experience in real-life settings about INSTIs resistance [15]. Moreover, there is limited data regarding the resistance pathways in patients failing INSTIs in the context of multi-experienced patients, where some shared resistance pathways between RAL and EVG, and cross-resistance pathways to DTG and bictegravir (BIC) are not well described.

In resource-limited settings (such as in Mexico and most countries in Latin America), where the overall use of integrase inhibitors has been increasing in recent years, data for predicting the viral response to INSTIs, when switching to another treatment is limited. This study aimed to investigate the resistance-associated mutations in the HIV-1 integrase gene and to evaluate the resistance and cross-resistance pathways to INSTIs in a cohort of ART multi-experienced Mexican patients failing to RAL-containing regimens.

## Methods

### Study design

The study was conducted at the Molecular Virology Unit of the Infectious Diseases Department of the Instituto Nacional de Ciencias Médicas y Nutrición Salvador Zubirán (INCMNSZ) in Mexico City, a national reference unit for HIV positive patients. A cross-sectional analysis of 25 plasma samples from HIV-1 infected adult subjects collected from various healthcare centres throughout the country from 2009 to 2017 for viral load testing (available from our samples bank) was conducted. Samples were selected from subjects failing a RAL-containing salvage regimen (defined as two consecutive HIV-1 viral loads above the limit of detection after suppressive ART or not achieving undetectable RNA viral load after 6 months of highly active antiretroviral therapy) at the time that the sample was collected. In all cases, the patients were naive to INSTIs before using RAL and their RAL-containing regimen were prescribed as third line ART. Antiretroviral treatment history and resistance history data available from subjects were also collected.

### Laboratory assays

HIV-RNA viral load (VL) was measured in all samples with the AmpliPrep/COBAS TaqMan HIV-1 Test, v2.0 (Roche Molecular Systems, Branchburg, NJ, USA); and CD4 + total cell count was measured by flow cytometry (BD Multitest™ CD3/CD8/CD45/CD4 in BD FACSCanto™ II, BD Biosciences, San Jose, CA, USA). Plasma samples stored at − 70 ℃ were used for resistance testing. The RNA viral extraction step was performed using a modified protocol of the ViroSeq HIV-1 Genotyping System (Celera Diagnostics, Alameda, CA, USA) using 1000 mL of plasma volume plus centrifugation at 17,000 rpm for 2 h, 4 ℃ temperature for viral precipitation. RNA elution was performed according to the viral load value; VL > 20,000 copies/mL, 30 mL of elution buffer; VL 1000–19,999 copies/mL, 20 mL elution buffer and < 1000 to 100 copies/mL, 15 mL of elution buffer. Amplification of the HIV-1 integrase gene region was performed either using the ViroSeq HIV-1 Integrase Genotyping System (Celera Diagnostics, Alameda, CA, USA) or our in-house standardized nested PCR strategy: A one-step reverse transcription and first round of PCR was conducted using the OneStep RT-PCR kit (QIAGEN, Hilden, Germany) with in-house designed primers, forward AOR-IN-F (5′-CAGTGCTGGAATCAGGAAAGTA-3′) and reverse AOR-IN-R (5′-CTTGGATGAGGGCTTTCATAGT-3′). PCR products from the first round were used as template for the second one that was performed using the AmpliTaq Gold DNA Polymerase with Buffer II (ThermoFisher, Foster City, CA, USA) and the primers INPS1 (5′-TAGTAGCCAGCTGTGATAAATGTC-3′) and INPR8 (5′-TTCCATGTTCTAATCCTCATCCTG-3′) from the ANRS AC11 Resistance Study Group protocol, available at: http://www.hivfrenchresistance.org/ANRS-procedures.pdf (Accessed November 11, 2015). The resulting PCR products were visualized and measured with the Agilent Bioanalyzer 2100 system and the DNA 7500 kit (Agilent Technologies, Waldbronn, Germany), products were purified with the Microcon Centrifugal Filters (Merck, Darmstadt, Germany) and prepared with the BigDye Terminator v3.1 (Applied Biosystems, Austin, TX, USA) for Sanger automated capillary sequencing using the 16-capillary 3130xl Genetic Analyzer (Applied Biosystems, Austin, TX, USA).

### Data analysis

Viral sequences were analysed with the SeqScape v2.5 software and the FASTA files were analysed using the Stanford HIV Drug Resistance Database v8.8 algorithm (http://hivdb.stanford.edu/), defining resistance interpretation of penalty scores as susceptible (score 0–9), potential low-level resistance (score 10–14), low-level resistance (score 15–29), intermediate resistance (score 30–59) and high-level resistance (score ≥ 60). The REGA HIV-1 Subtyping Tool version 3.0 (http://dbpartners.stanford.edu:8080/RegaSubtyping/stanford-hiv/typingtool/) was used for the HIV subtype analysis.

Descriptive statistics, proportions and dispersion measures (with standard deviation) were described for population features, antiretroviral treatment history variables and laboratory results. Data were analysed using the IBM© SPSS© Statistics v23.0.0.0 software. Correlation and Chi square analysis were performed. A P < 0.05 was considered significant.

## Results

### Study population characteristics

Twenty-five plasma samples from 20 (80%) male subjects and 5 (20%) female subjects fulfilling eligible criteria (from 2009 to 2017) were successfully genotyped and included for the analysis. The mean duration of treatment prior to current failure was 22.33 months (SD 20.38, *n *= 15) while the mean time under ART was 10.65 years (SD 5.7, *n *= 12). Subjects had been exposed to a mean of 5.31 (SD 3.13, *n *= 16) ART combinations.

The HIV-1 subtyping analysis of the sequences indicated subtype B for all 25 samples. At RAL failure, the mean viral load was 4.17 log^10^ copies/mL (SD 1.11, *n *= 22) and the mean of CD4 + T-cell count was 328.5 cells/mm^3^ (SD 217.07, *n *= 12) (Table 1).

**Table 1:** Study population characteristics

Feature		*n*
Sex, male (%)	20/25 (80%)	25
HIV-1 subtype B (%)	25/25 (100%)	25
Plasma HIV-1 RNA, log^10^ copies/mL [mean (SD)]	4.17 (1.11)	22
CD4 + T-Cell count, cells/mm^3^ [mean (SD)]	328.5 (217.07)	12
RAL exposure time, months [mean (SD)]	22.33 (20.38)	15
Time on ART, years [mean (SD)]	10.65 (5.7)	12
Total prior ART regimens [mean (SD)]	5.31 (3.13)	16
Optimized backbone regimen (%)		17
NRTIs	5/17 (29.41%)	17
NRTIs + PI	5/17 (29.41%)	17
NNRTIs + PI	3/17 (17.65%)	17
NRTIs + NNRTIs	1/17 (5.88%)	17
Other^a^	3/17 (17.65%)	17

*SD* standard deviation, *NRTIs* nucleoside reverse transcriptase inhibitors, *PI* protease inhibitor, *NNRTIs* non-nucleoside reverse transcriptase inhibitors, *ART* antiretroviral therapy

^a^Regimens including enfuvirtide. N = 25

The most common ART combinations included NRTIs (Table 1) either alone or with a protease inhibitor (PI). Overall, tenofovir (TDF) was the most frequent drug across the subjects (58.82%, *n *= 17).

### Integrase resistance patterns

**Fig. 1:**
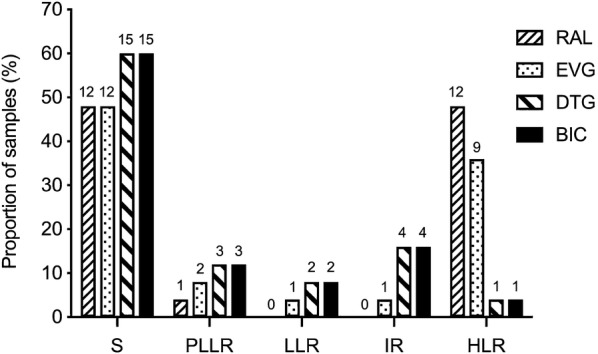
Proportion of samples with genotype-predicted resistance to integrase strand-transfer inhibitors. Absolute numbers are shown above each bar. *RAL* raltegravir, *EVG* elvitegravir, *DTG* dolutegravir, *BIC* bictegravir, *S* Susceptible (score 0–9), *PLLR* Potential low-level resistance (score 10–14), *LLR* Low-level resistance (score 15–29), *IR* Intermediate resistance (score 30–59), *HLR* High-level resistance (score ≥ 60); N = 25

The number of samples with genotype-predicted resistance (susceptibility score ≥ 15) to RAL was 12/25 (48%), while 11/25 (44%) were resistant to EVG and 7/25 (28%) to both DTG and BIC (Fig. 1).

Thirteen out of the 25 samples (52%) were found to be fully susceptible to the four INSTIs (susceptibility score < 15). Of the RAL-resistant samples (*n *= 12), cross-resistance to EVG was found in 10/12 (83.3%) while 2/12 samples (16.7%) had resistance to DTG and BIC.

### Integrase inhibitors RAMs

**Fig. 2:**
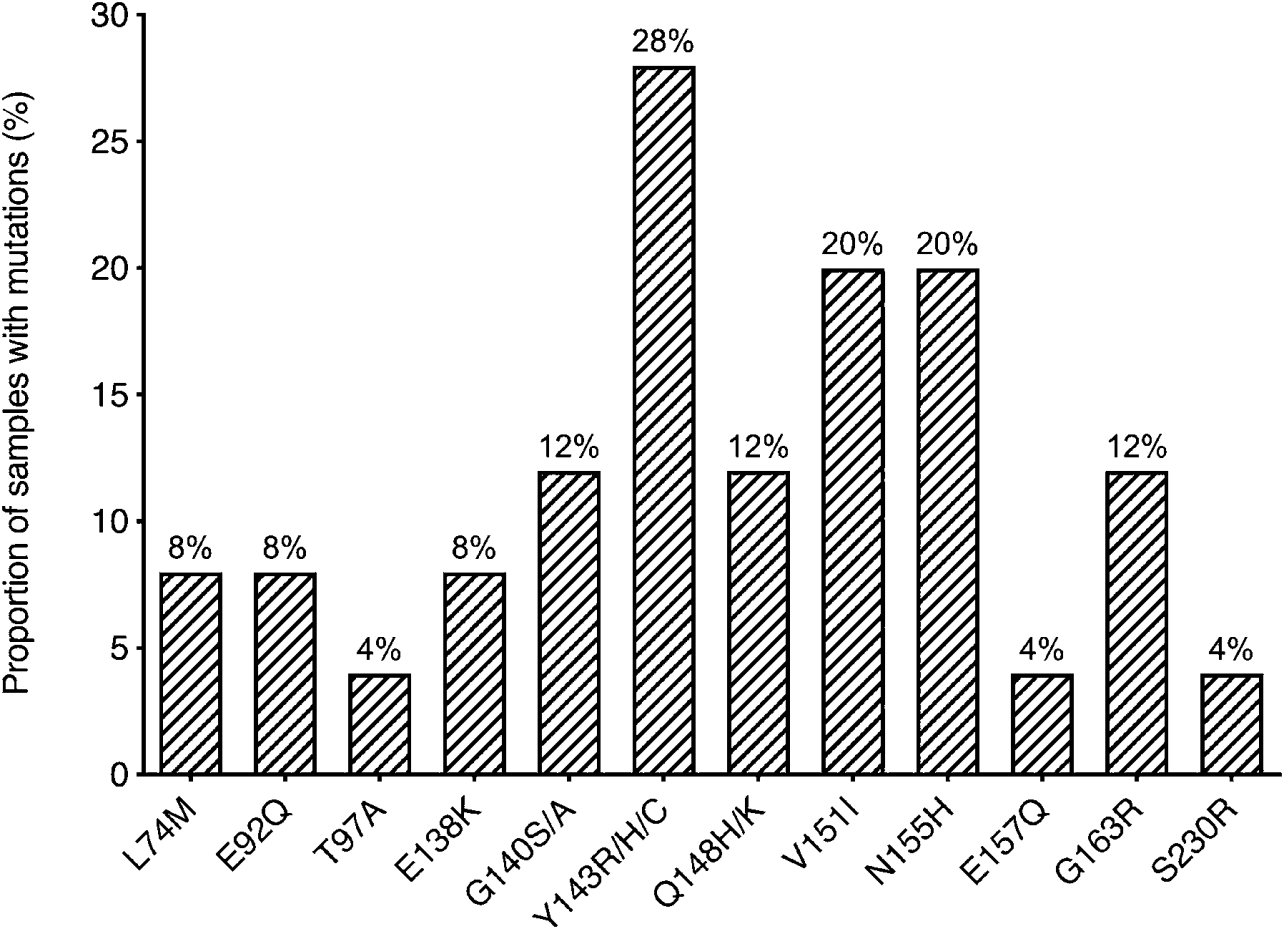
Frequencies of samples with integrase resistance-associated mutations. *INSTIs* integrase strand transfer inhibitors, *RAMs* resistance associated mutations; N = 25

Concerning integrase RAMs, changes at the Y143 position were the most frequent (Fig. 2). Overall, the non-polymorphic integrase mutations occurred at positions; Y143 (28%), V151 (20%), N155 (20%), Q148 (12%), G140 (12%), G163 (12%), L74 (8%), E92 (8%), E138 (8%), T97 (4%), E157 (4%) and S230 (4%).

The Q148 + G140 combination of substitutions, which has been related to DTG resistance [10, 15] was observed in 3/25 samples (12%) (subjects 5, 8 and 14) as shown in Table 2.

**Table 2:** Resistance-associated mutations (RAMs) and other substitutions found in the integrase region along with the susceptibility score

Subject	Major	Accessory	Other	Score RAL	Score EVG	Score DTG	Score BIC
1	Y143R	T97A	L101I, V201I, S230N	70	25	5	5
2	–	–	K111R, T125A, T206S, S230N, D232E	0	0	0	0
3	–	–	I72V, L101I, T124A, V126T, I135V, F181L, V201I, K215N, D232E	0	0	0	0
4	–	–	A80V, L101I, T112V, T124N, T125V, I141V, G163E, V201I, I203M	0	0	0	0
5	G140S, Q148H	–	L101I, T112A, T124A, T125A, I135V, K156N, D256E	90	90	45	45
6	E92Q, E138K, Y143HR	–	L68V, L74M, S119T, V201I, S230N, D232E, L234F	105	90	30	30
7	–	–	L101I, K111R, T112S, R187K	0	0	0	0
8	G140S, Y143R, Q148H	–	L101I, K160N, L234I	150	100	50	50
9	–	–	I113V, T124N, T125A, K211R, K219N, N222K, D232E, D253E	0	0	0	0
10	–	–	V31I, M50L, L101I, T112I, V201I	0	0	0	0
11	–	–	D25E, V31I, I72V, Y99F, L101I, T124A, T125A, I135V, G163T	0	0	0	0
12	Y143H	–	F1I, D3V, V31I, Q44P, I72V, I73T, L102FPS, G106A, S230N, D256E	60	10	5	5
13	–	E157Q	S17N, A21E, A23V, L28I, P30A, S39C, I113V, T124A, T125A, K160R, V201I, L234I, W243G, D253E, S255N	10	10	0	0
14	E138K, G140A, Q148K	–	V31I, I72V, K111R, V201I, I208M, K215N, I220L	120	120	80	80
15	–	–	L101I, K136N, V151I, V201I, T206S, E212V, K215N, D256E, S283G	0	0	0	0
16	Y143R	G163R	V31I, L74M, Y99F, G106A, T112A, I113V, S123V, T124A, T125A, I204V, S230N, K240R	75	35	15	15
17	E92Q, N155H	G163R	V31I, L101I, T112AIV, T124A, E138D, V151I, K211R	105	135	25	25
18	–	–	S24N, T124A, K160Q, V201I, S230N	0	0	0	0
19	Y143C, N155H	S230R	K14R, V31I, E69Q, Y99F, L101I, S119T, T124A, I135V, G163E, K211R, L234V, D256E	140	95	40	30
20	N155H	G163R	T112A, T124A, I135V, V151I, K156R, V201I, I208M	75	75	10	10
21	N155H	–	I72V, L101I, S119R, V151I, K156N, I208L, E212L, T218S	60	60	10	10
22	–	–	L68M, T122S, T124A, V151I, G193E, I208L	0	0	0	0
23	N155H	–	L101I, K111T, S119T, T124A, T125A, I208L	60	60	10	10
24	–	–	L101I, K111T, I113V, S119P, K211R, K219N, N222K, S230N	0	0	0	0
25	–	–	I72V, I73V, A80V, L101I, K111R, T124A, E138D, G163V, G197W, R228K	0	0	0	0

Stanford HIV Drug Resistance Database v8.8. N = 25

*RAMs* resistance-associated mutations, *RAL* raltegravir, *EVG* elvitegravir, *DTG* dolutegravir, *BIC* bictegravir

Also, we found the L101I/T124A combination in 7/25 samples (28%) (subjects 3, 5, 11, 17, 19, 23 and 25). Four of these samples (subjects 3, 11, 23 and 25) were fully susceptible to INSTIs. Two out of four samples with intermediate DTG resistance had harboured such combination (subjects 5 and 19). The DTG resistance associated mutation R263K was not found. However, one sample (subject 13) had the presence of the E157Q substitution (Table 2).

We did not find any association or significant correlation of the presence of drug resistance and any of the mutations with the baseline characteristics of our study population. Data about adherence was not entirely available, and therefore it was not analysed.

### Susceptibility of the RAL-containing regimen

Only 17 out of the 25 subjects had fully documented treatment history available. From these 17, only 11 (64.7%) had a regimen with ≥ 2 susceptible drugs (including RAL). Also, 11 out of those 17 (64.7%) had mutations in the reverse transcriptase and protease regions (RT-PR) that were affecting the current antiretroviral regimen, 2/17 samples (17.6%) had mutations that were not affecting the susceptibility of the regimen and 2/17 samples (17.6%) had no mutations.

Among the samples that were fully susceptible to all INSTIs, 6/9 (66.7%) were also susceptible to their respective RT-PR backbone regimen. None of these subjects had enfuvirtide (T20) in their regimen. RT-PR drug resistance mutations of the samples and their backbone regimen are summarized in Table 3.

**Table 3:** Susceptibility of the regimen and RT-PR associated resistance mutations

Sample^a^	Backbone regimen	RT mutations	PR mutations	Susceptible drugs (including RAL)^b^
2	ETV + DRV/r	–	–	3
3	TDF + ZDV + LPV/r	–	–	3
6	TDF/FTC	M184V, V179D	–	2
7	TDF + DRV/r	K103N, Y318F	–	3
8	ETV + DRV/r + T20	E44D, L210W, T215D, K103N, E138G, V179E	V32I, M46I, I54L, V82T, I84V, L33F, T74P	2
11	TDF/FTC + ETV	–	–	4
12	TDF + ZDV + T20	K103N, V108I, H221Y	–	4
13	TDF/FTC + DRV/r	M41L, L74V, M184V, L210W, T215Y, K103S, G190A, F227L	M46L, I54V, I84V, L24M, Q58E	1
14	TDF/FTC	M41L, M184V, T215Y, K103N, K238T	–	1
17	ABC/3TC	M184V	–	1
18	ETV + DRV/r	M184V, K103N, M230L	L33F, M46I, I47V, I54M, V82M	1
19	TDF/FTC	M41L, E44D, D67N, L74V, M184V, L210W, T215Y, K219E, L100I, K103N	L90M	2
21	TPV/r + T20	M41L, A62V, T69D, V75I, Y115F, F116Y, Q151M, M184V, L210W, T215Y, K219R, L100I, K103S, F22FL	V32I, M46I, I54V, V82F, I84V, L90M, L33F, K43T, T74P	2
22	ABC/3TC	M184MIV	–	1
23	TDF + LPV/r	M41L, T215Y	M46I, I54V, V82A, L24I	1
24	TDF/FTC + ATV/r	D67N, T215A, K219Q	–	3
25	EFV + DRV/r	M41L	–	3

*RT* reverse transcriptase, *PR* protease, *ETV* etravirine, *DRV/r* darunavir and ritonavir, *TDF* tenofovir, *ZDV* zidovudine, *LPV/r* lopinavir and ritonavir, *FTC* emtricitabine, *T20* enfuvirtide, *ABC* abacavir, *3TC* lamivudine, *TPV/r* tipranavir and ritonavir, *EFV* efavirenz

^a^Samples from subjects with backbone resistance data available

^b^Number of drugs with a predicted resistance score < 15 (T20 was considered susceptible for all cases). *n *= 17

## Discussion

The antiretroviral therapy for HIV infection should not be left out from antimicrobial resistance stewardship strategies. In this regard, the World Health Organization developed a global strategy for the prevention and assessment of HIV drug resistance to support an approach for monitoring and surveillance in public health [16, 17]. We want to highlight the importance of performing resistance surveillance especially for INSTIs, drugs that will be used with worldwide. So far, multi-experienced patients failing integrase inhibitors are difficult to find in our settings. Nevertheless, they still can be found in the HIV services, and drug resistance is one of the major challenges for clinicians who treat them. Our subjects were heavily ART experienced, and so their ART salvage options were somewhat limited by the resistance data.

Despite the concentration of the HIV epidemic in Mexico among men who have sex with men (MSM) and other key populations [18], we found that 5 of our 25 samples were from female subjects, highlighting that this demographic group should not be underestimated in our setting.

The genetic analysis exhibited HIV subtype B strains for all samples. This is consistent with epidemiological data in Latin America that describes subtype B as the most prevalent subtype in the region [19]. We would like to further investigate integrase resistance patterns across different subtypes.

Forty-eight percent of our samples had resistance to RAL while the other (52%) were still susceptible. This absence of detectable resistance after treatment failure in our samples might have been the result of the presence of drug-resistant minority viral populations, a prolonged interval between the time of antiretroviral drug discontinuation and genotypic testing, poor adherence and/or drug–drug interactions (leading to sub-therapeutic drug levels), although, none of these causes could be fully documented in our study. Supporting the likely absence of selective pressure and the lower occurrence of resistance, is the comparison that our data with the BENCHMRK 1 and 2 trials that included similar cases to ours [20]. We found a lower frequency (48%) of patients who had integrase mutations at important residues (Y143, Q148 or N155) compared with the 65% reported in these studies [20].

As we suspected, cross-resistance to EVG was higher than cross-resistance to the other INSTIs in our study. The presence of the N155H substitution (one of the second most frequent in our study) reduces both RAL and EVG susceptibility but does not, alone, compromise DTG nor BIC by itself (thus, having more resistance to RAL leads to more EVG cross-resistance).

We found a similar proportion of DTG resistance (28%) to the 35% reported in the study of Cavalcanti et al. conducted in Brazil with patients failing RAL [21]. Both mean CD4+ cell count and viral load from our subjects at the time of resistance testing were very similar to the ones reported in that study (CD4+ cell count of 221 cells/mm^3^ and 3.99 log^10^ copies/mL of viral load). Those cases were exposed to RAL for a mean of 115 weeks with prior use of 7 regimens while our patients had 89.32 weeks (22.33 months) of RAL exposure and a mean of 5.3 regimens. On the other hand, we do not find a significant difference between the mean viral load of the subjects with DTG resistance compared to the subjects without DTG resistance (*n *= 22), nor with samples harbouring a G140S/A + Q148H/R/K combination (*n *= 3) as described in that study. This is important since the presence of the G140S mutation in combination with the Q148H mutation has been described to improve the viral fitness and increase the viral load in RAL-resistant subjects [22].

According to our findings, the frequency of the substitution Q148+ ≥1 secondary mutation(s) (G140A/C/S, L74I or E138A/K/T) was 12%. These combinations were found to reduce the response to DTG after RAL expose in the VIKING-3 study [15] (Table 4), while here, the proportion of patients without INSTIs mutations was higher. Drug resistance testing is recommended when DTG (and potentially BIC) is considered for salvage therapy.

**Table 4:** Comparison to RAMs combinations decreasing response to dolutegravir in the VIKING-3 study

RAMs combinations after RAL use	Prevalence (%)	
	VIKING-3 [15]	This study
No INSTIs mutations	33	48
No Q148^a^	36	40
Q148 + 1 secondary mutation^b^	20	8
Q148 + ≥2 secondary mutations^b^	11	4

*RAMs* resistance associated mutations, *RAL* raltegravir, *INSTIs* integrase strand transfer inhibitors

^a^Y143, N155, T66 or E92

^b^G140A/C/S, L74I or E138A/K/T

Sequencing has demonstrated non-polymorphic mutations and combinations associated with INSTIs exposure, which do not, alone, cause resistance. The L101I/T124A which may vary between HIV subtypes as reported by Garrido et al. was more frequently observed in our cohort compared to theirs (for subtype B) [23]. Also, we found the L101I/T124A combination at a higher frequency in our cohort (28%) than that reported by Saladini et al. (10.8%) for RAL experienced patients [24]. This combination has been more frequently described in RAL experienced patients and has been suggested to play a role in the modulation of response to DTG in vitro by favouring the selection of other mutations [25].

The E157Q substitution (found in subject 13) is a natural polymorphism described as a compensatory mutation for R263K-mediated DTG resistance [26] and after RAL exposure [27]. More information is needed to further recommend the use of DTG in naive patients harbouring the E157Q substitution as suggested by Charpentier and Descamps [28].

This study was limited by incomplete information about; treatment history, adherence, the HIV-1 viral load and CD4+ T-cell count. Before-mentioned data, could not be located due to loss of retention in care, the transfer of care centre or the passing of the subjects. The follow-up of the subjects was also limited and clinical outcomes could not be fully described. Moreover, RAMs in the RT-PR region illustrates the high exposure to previous ART regimens. On the other hand, the absence of mutations in 66.7% of the subjects (for RT-PR and integrase regions) may be explained by poor adherence and lower drug exposure. However, we cannot exclude a significant role for minority variants, which should be further investigated.

## Conclusions

This is the first study to explore the integrase inhibitors resistance profiles in ART multi-experienced subjects in our country. RAL resistance was documented in 48% of the samples. Predicted cross-resistance to EVG was very high as described in clinical trials, while cross-resistance to DTG was limited (83.33% and 18.18% respectively). The frequencies of INSTIs RAMs at RAL failure were lower than those reported in the BENCHMRK 1 and 2 studies [20]. On the other hand, the occurrence of DTG cross-resistance was also lower than that reported in the VIKING-3 study [15]. These lower proportions could be related to poor adherence or to the genetic barrier of the overall ART combinations.

We found a high frequency of the polymorphic combination L101I/T124A than that reported for subtype B strains. This combination has been suggested to be somehow related to DTG resistance and is more prevalent in RAL experienced patients in comparison to RAL-naive patients [25].

The RAL non-resistant samples were still susceptible to all INSTIs. However, it is difficult to support the continuation of RAL in those cases without strong evidence from controlled clinical trials. The role of minor viral strains and other regions outside the integrase gene should also be investigated especially in patients without documented RAMs.

In summary, we found an important occurrence of RAL drug resistance and cross-resistance in our cohort. The relationship between specific clinical features and the development of drug resistance in multi-experienced subjects should be further investigated. Given that multi-experienced patients failing integrase inhibitors are difficult to find, our cohort represents an important number of real-life cases. Furthermore, the number of people with INSTIs-based therapy will be growing in Latin-America following recent changes in international and local guidelines. The decision of using DTG (or potentially BIC) for salvage therapy in the presence of RAL failure or suspected resistance to INSTIs should be made following an integrase resistance test. Despite the similar profiles of DTG and BIC resistance, more information in real-life settings will be needed to guide future salvage regimens. Moreover, we urge reinforcing adherence as well as close surveillance in multi-experienced patients to prevent the emerging of resistance and likely transmitted resistance to integrase inhibitors.

## Data Availability

Integrase sequences have been submitted to GenBank and are available under accession numbers MF154570 to MF154594.
